# The impact of Pegylated liposomal doxorubicin in recurrent ovarian cancer: an updated meta-analysis of randomized clinical trials

**DOI:** 10.1186/s13048-021-00790-4

**Published:** 2021-03-09

**Authors:** Xin-Ru Li, Yi Zhu, Guo-Nan Zhang, Jian-Ming Huang, Li-Xia Pei

**Affiliations:** 1 Hospital of Chengdu University of Traditional Chinese Medicine, Chengdu, 610072 Sichuan Province People’s Republic of China; 2 Department of Ultrasound, the Affiliated Cancer Hospital, School of Medicine, University of Electronic Science and Technology of China, Sichuan Cancer Hospital & Institute, Chengdu, 610041 Sichuan Province People’s Republic of China; 3 Department of Gynecological Oncology, the Affiliated Cancer Hospital, School of Medicine, University of Electronic Science and Technology of China, Sichuan Cancer Hospital & Institute, No.55 Ren-min-nan Road, Chengdu, 610041 Sichuan Province People’s Republic of China; 4 School of Clinical Medicine, Chengdu University of Traditional Chinese Medicine, No.37 Shi-er-qiao Road, Chengdu, 610072 Sichuan Province People’s Republic of China; 5 Department of Biochemistry & Molecular Biology, the Affiliated Cancer Hospital, School of Medicine, University of Electronic Science and Technology of China, Sichuan Cancer Hospital & Institute, Chengdu, 610041 Sichuan Province People’s Republic of China

**Keywords:** Ovarian neoplasms, Pegylated liposomal doxorubicin, Progression-free survival, Overall survival, Meta-analysis

## Abstract

**Background:**

Previous meta-analysis studies suggested that pegylated liposomal doxorubicin (PLD) may improve the survival rate of patients with recurrent ovarian cancer. The aim of the present meta-analysis, then, was to further update the role of PLD in the treatment of recurrent ovarian cancer.

**Methods:**

We performed a literature search using the electronic databases Medicine, EMBASE, Web of Science, and the Cochrane Library to 27 July 2020. We only restricted the randomized clinical trials. Study-specific hazard ratios and 95% confidence interval (HR/95% CI) and risk ratios and 95% confidence interval (RR/95% CI) were pooled using a random-effects model.

**Results:**

Ten studies (12 trials) were included after screening 940 articles. We categorized the eligible studies into two groups: the doublet regimens (four trials, 1767 patients) showed that PLD plus carbo provided superior progression-free survival (PFS) (HR, 0.85; 95% CI, 0.74–0.97) and similar overall survival (OS) (HR, 1.00; 95% CI, 0.88–1.14) compared to paclitaxel (PAC) plus carboplatin (carbo). PLD plus carbo was associated with significantly more anemia and thrombocytopenia, and other side effects were well tolerated. The monotherapy regimens (eight trials, 1980 patients) showed that PLD possessed a similar PFS (HR, 1.02; 95% CI, 0.90–1.16) and OS (HR, 0.88; 95% CI, 0.77–1.01) relative to other monotherapies. PLD alone was also more associated with mucositis/stomatitis and hand-foot syndrome, while other side effects were well tolerated.

**Conclusions:**

In platinum-sensitive recurrent ovarian cancer, PLD plus carbo was more effective than PAC plus carbo, while in platinum-resistant or -refractory recurrent ovarian cancer, PLD exhibited similar survival to other monotherapies. Regarding side effects, PLD plus carbo and mono chemotherapy were both well tolerated.

**Supplementary Information:**

The online version contains supplementary material available at 10.1186/s13048-021-00790-4.

## Introduction

Ovarian cancer is one of the most common gynecologic malignancies, with the third highest incidence of gynecologic tumors and the highest mortality rate. Because ovarian cancer is not easy to detect at the early stages, it is usually diagnosed at an advanced stage, and its 5-year relative survival rate is comparatively low. The lifetime risk for ovarian cancer is approximately 1 in 75, and the likelihood of dying from this malignancy is 1 in 100 [1, 2]. Cytoreductive surgery followed by platinum-based chemotherapy remains the mainstay of treatment in ovarian cancer. Yet, despite complete remission through the very best treatments, approximately 70–80% of patients with International Federation of Gynecology and Obstetrics stage III to IV disease experience a relapse within 5 years [3, 4]. Thus, ovarian cancer remains a serious threat to women’s health worldwide.

For patients with platinum-sensitive recurrent ovarian cancer, we usually choose carboplatin (carbo) in combination with paclitaxel (PAC) as the first-line standard chemotherapy regimen, but this regimen exhibits more non-hematologic toxicity, which results in early discontinuation of treatment. Specifically, this regimen imposes high rates of alopecia, hypersensitivity, and neurotoxicity [5], and platinum re-challenge therapy in platinum-refractory or -resistant patients usually results in low response rates and short survival. In this particular setting, chemotherapy with single agents shows activity and lower toxicity than combination chemotherapy [6]. Single-agent second-line treatments include non-platinum compounds such as PAC, topotecan, PLD, gemcitabine, etoposide, vinorelbine, and bevacizumab, and we typically choose sequential single chemotherapeutic agents depending upon the various conditions exhibited by patients. While treatment options for recurrent ovarian cancer have increased, the majority of these patients will still eventually die from ovarian cancer. Therefore, the goal of therapy in the recurrent setting should not only focus on improving the length of life but also include a thoughtful review of anticipated side effects and overall quality of life.

PLD—anthracycline chemotherapy derived from doxorubicin—was the first FDA-approved cancer nanomedicine [7], and was used as early as 2014 for the treatment of ovarian and breast cancer, multiple myeloma, and Kaposi sarcoma [8]. The 2017 NCCN Guidelines recommended that carbo combined with PLD be added as one of the initial chemotherapy regimens for ovarian cancer. Carbo combined with PLD was thus recommended for patients with recurrent platinum-sensitive ovarian cancer, and PLD monotherapy was recommended for relapsed platinum-resistant ovarian cancer patients. The 2018 NCCN Guidelines included PLD as a first-line chemotherapy regimen for ovarian cancer, and a regimen of carbo combined with PLD is recommended for initial treatment of stage-1 ovarian cancer. The 2019 NCCN Guidelines recommend that PLD plus bevacizumab be used as a potential treatment option for patients with platinum-resistant recurrent ovarian cancer. Clinical studies have shown that compared with other standard chemotherapy regimens, PLD possesses a non-inferior survival rate and is well tolerated, exhibiting reduced alopecia and neurotoxicity [9].

Previous studies [10, 11] showed that PLD is effective and well tolerated in the treatment of ovarian cancer. However, because these two meta-analyses were published earlier and contained fewer trials, we added the most recent trials and performed an updated meta-analysis. We trust that our study results will soon facilitate the selection of chemotherapy regimens for recurrent ovarian cancer patients.

## Methods

### Search strategy

We conducted this meta-analysis framework under the guidance of PRISMA, and performed queries of the literature using the electronic databases Medicine, EMBASE, Web of Science, and the Cochrane Library to 27 July 2020. The search MeSH terms and free words used were 1) “Pegylated Liposomal Doxorubicin,” “Caelyx,” “Lipodox,” “Doxil,” 2) “ovarian cancer,” “ovarian neoplasm,” “ovarian carcinoma,” and 3) “Randomized Controlled Trial.” We did not limit the language for our searches or the studies included in the present investigation. The details of the search strategy are presented in Supplementary Material 1.

### Eligibility criteria

The abstracts of all articles retrieved in the initial search were independently screened by two authors (X.R.L and L.X.P). The procedures were executed by the independent reviewers according to the following criteria. The inclusion criteria were 1) patients with histologically confirmed recurrent ovarian cancer; 2) patients with interventions involving PLD alone versus other monotherapy, or PLD plus carboplatin versus paclitaxel plus carboplatin; 3) outcome measures that involved survival outcome and adverse events; and 4) all RCT studies. The exclusion criteria were 1) patients not having previously received PLD; 2) patients not having undergone any examinations for ovarian cancer; 3) pediatric populations (< 18 years of age); 4) animal/laboratory studies; 5) review articles, case reports, letters, commentaries, or conference proceedings; and 6) no histologic confirmation of recurrent ovarian cancer. Disagreements were discussed with a third author (Prof. G.N.Z) to achieve consensus.

For the present study, the same two authors who performed full-text screening independently conducted data extraction, and all inconsistencies were resolved by consensus. Selected full-text manuscripts were reviewed in detail to determine their relevance. The exclusion criteria were 1) those studies not within the current research aims; 2) studies with missing data; and 3) overlapping studies.

### Data extraction

Data were extracted from the studies that we ultimately used, and data included first author, journal, year of publication, number, age and characteristics of patients, study design, and outcomes.

### Statistical analysis

For survival variables such as progression-free survival (PFS) and overall survival (OS), we used hazard ratios (HR) and 95% CI, which are presented as forest plots. For categorical variables, we used risk ratios (RR) and 95% CI, which are also presented as forest plots. Heterogeneity across studies was evaluated using the I^2^ metric and Chi-squared test. We used the random-effects model to calculate the summary estimate if heterogeneity was shown (I^2^ > 50%) across studies; otherwise, the fixed-effects model was used (I^2^ ≤ 50%). If heterogeneity was uncovered across studies, we performed subgroup analyses based upon study design and then analyzed the subgroup results. If potential publication bias was shown across studies, we used Egger’s linear regression test, as well as Begg’s funnel plot. All statistical testing was conducted using the Review Manager 5.3 (Copenhagen, The Nordic Cochrane Centre, The Cochrane Collaboration, 2014) and Stata.15.0 (Stata-Corp, College Station, TX). All tests were two-sided with *P* < 0.05 considered statistically significant, except for the heterogeneity test (*P* < 0.1) and publication bias (*P* < 0.1) in our meta-analyses.

## Results

### Literature search

**Fig. 1:**
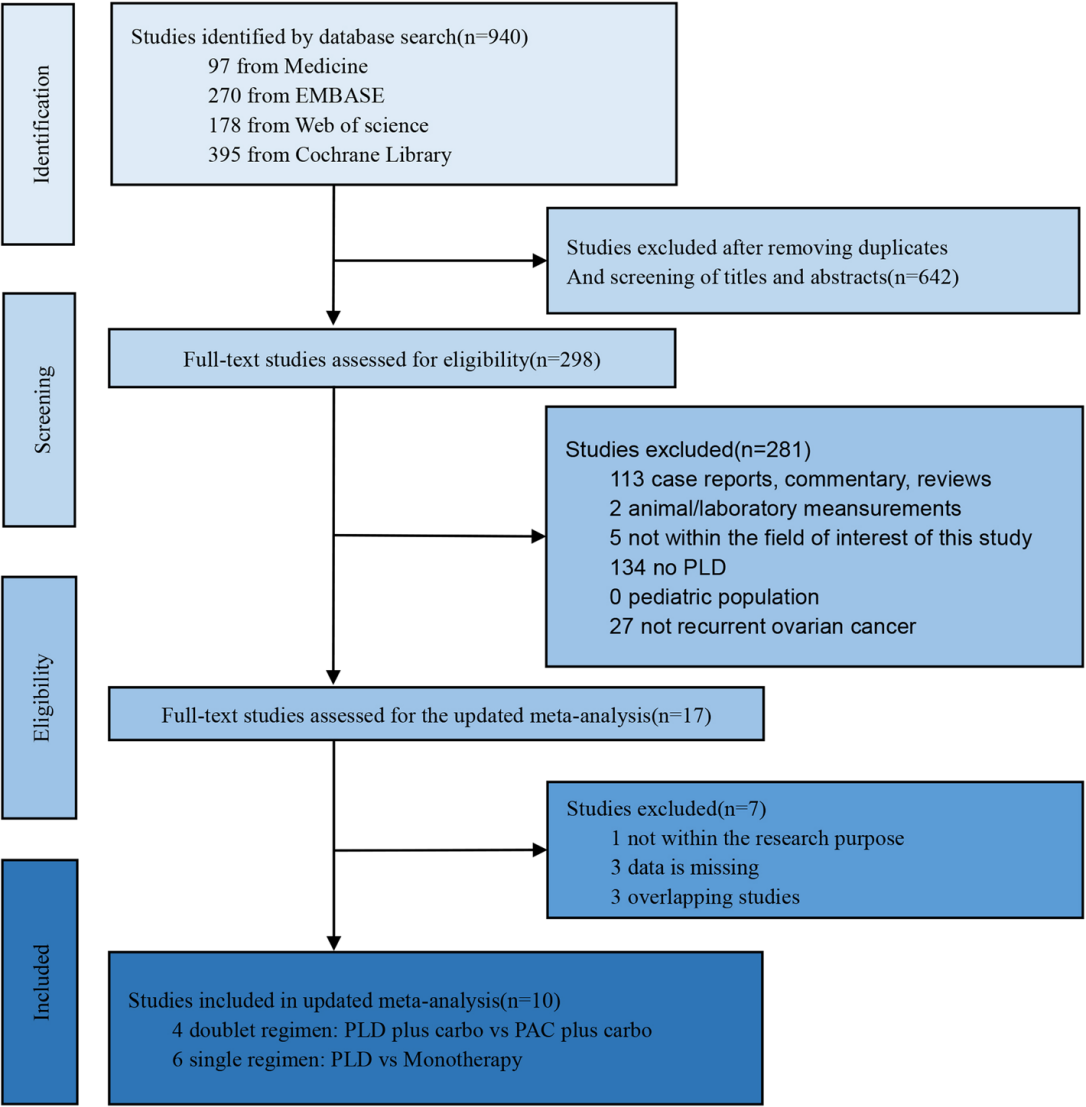
Flow diagram showing selection of the studies for this updated meta-analysis

We designated for initial evaluation a total of 940 articles using our electronic database search. After removing duplicate articles and screening the study titles and abstracts, 56 articles meeting the inclusion criteria underwent full-text assessment, resulting in 10 relevant studies [12–22]. A flowchart of the selection procedure is shown in Fig. 1.

### Study characteristics

We categorized the 12 eligible trials into two groups: PLD plus carbo vs. PAC plus carbo (four trials [12–15]: 851 PLD plus carbo and 916 PAC plus carbo), and PLD vs. other monotherapies (eight trials [16–21]: 963 PLD and 1017 other monotherapies). Vergote 2009 [18] was utilized in both trials—PLD vs topotecan and PLD vs canfosfamide, and Kaye 2012 [21] was integrated into both trials—PLD vs. 200 mg of olaparib and PLD vs. 400 mg of olaparib. All features of the included studies are depicted in Table.1. We assessed the study quality based on the Cochrane Collaboration tool and the criteria specified in Chapter 8 of the Cochrane Handbook for Systematic Reviews of Interventions [23] (Table 2). Each study was evaluated for potential bias and quality by two independent and experienced authors, and disagreements were resolved by consensus.

**Table 1:** Characteristics of included studies

Study	Intervention	No.of participants	Age,years Median (range)	Type of trial	Patient characteristics	Pretreatment status	Main outcomes		
Pujade-Lauraine 2010 [13]	carbo(AUC5) + PLD 30 mg/m2 q4wks	466	60.5 (24–82)	phase III randomized multicenter, open-label trial	PS ROC	After first-or second-line Platinum and taxane-based	PFS, OS, Toxicity		
	carbo(AUC5) + PAC 175 mg/m2 q3wks	509	61 (27–82)						
Gladieff2012 [14]	carbo(AUC5) + PLD 30 mg/m2 q4wks	161	60 (24–82)	phase III randomized non-inferiority trial	PS ROC	After first- or second-line platinum- and taxane-based	PFS, Toxicity		
	carbo(AUC5) + PAC 175 mg/m2 q3wks	183	60 (30–80)						
Mahner2014 [15]	carbo(AUC5) + PLD 30 mg/m2 q4wks	131	60 (30–80)	phase III randomized multicenter trial	PS ROC	Platinum and taxane-pretreated	PFS, OS, Toxicity		
	carbo(AUC5) + PAC 175 mg/m2 q3wks	128	63 (27–82)						
Bafaloukos2010 [16]	carbo(AUC5) + PLD 45 mg/m2 q4wks	93	62 (38–89)	phase II randomized multicenter	PS ROC	One cycle or more Of platinum-based	ORR, OS, Toxicity		
	carbo(AUC5) + PAC 175 mg/m2 q3wks	96	63 (37–81)						
Mutch2007 [17]	PLD 50 mg/m2 IVI q4wks	96	62 (28–83)	phase III randomized multicenter open-label	PS ROC	Prior platinum-based ≤ 2 prior regimens allowed	PFS, OS, Toxicity		
	Gemcitabine 1000 mg/m2 D1,8 q3wks	99	59 (38–85)						
Ferrandina2008 [18]	PLD 40 mg/m2 IVI q4wks	76	63 (28–80)	phase III randomized multicenter	Partial PS and PR ROC	Failed first-line Platinum or paclitaxel	OS, Toxicity		
	Gemcitabine 1000 mg/m2 D1,5,8,15 q4wks	77	63 (39–79)						
Vergote2009I [19]	PLD 50 mg/m2 IVI q4wks	130	60 (30–82)	phase III randomized multicenter	platinum-refractory or PR ROC	Failed one second-Line therapy with either topotecan or PLD	Toxicity		
	Canfosfamide 1000 mg/m2 q3wks	231	60 (26–85)						
Vergote2009II [19]	PLD 50 mg/m2 IVI q4wks	130	60 (30–82)	phase III randomized multicenter	platinum-refractory or PR ROC	Failed one second-Line therapy with either topotecan or PLD	Toxicity		
	Topotecan 1.5 mg/m2 D1–5 q3wks	87	60 (30–82)						
Colombo2012 [20]	PLD 50 mg/m2 IVI q4wks	417	59 (23–84)	phase III randomized open-label	PR ROC	Failed≥4 cycles of platinum-based or discontinued	PFS, OS, Toxicity		
	Patupilone 10 mg/m2 IVI q3wks	412	59 (25–87)						
Banerjee2018 [21]	PLD 40 mg/m2 IVI q4wks	48	62 (52–86)	phase II randomized open-label	PR ROC	Progressed or relapsed < 6 months with a platinum-based	PFS, Toxicity		
	LIFA 2.4 mg/kg q3wks	47	62 (43–83)						
Kaye2012I [22]	PLD 50 mg/m2 IVI q4wks	33	53 (43–81)	phase II open-label randomized Multicenter	Partial PS and PR ROC	Recurred or progressed < 12 months with platinum-based	PFS, OS, Toxicity		
	Olaparib 200 mg bid continuously	32	58.5 (45–77)						
Kaye2012II [22]	PLD 50 mg/m2 IVI q4wks	33	53 (43–81)	phase II open-label randomized Multicenter	Partial PS and PR ROC	Recurred or progressed < 12 months with platinum-based	PFS,OS, Toxicity		
	Olaparib 400 mg bid continuously	32	53.5 (35–76)						

Note: *OS* Overall survival; *PFS* Progression-free survival; *PS* Platinum-sensitive; *PR* Platinum-resistant; *ROC* Recurrent ovarian cancer

**Table 2:** Risk of bias for included studies

Study	Random sequence generation	Allocation concealment	Blinding of participants and personnel	Blinding of outcome assessment	Incomplete outcome data	Selective reporting	other bias
Pujade-Lauraine2010 [13]	Low risk	Low risk	High risk	Low risk	Low risk	Low risk	Low risk
Gladieff2012 [14]	Low risk	Unclear risk	High risk	Low risk	Low risk	Low risk	Low risk
Mahner2014 [15]	Low risk	Low risk	High risk	Low risk	Low risk	Low risk	Low risk
Bafaloukos2010 [16]	Low risk	Low risk	Unclear risk	Low risk	Low risk	Low risk	Low risk
Mutch2007 [17]	Low risk	Low risk	High risk	Unclear risk	Unclear risk	High risk	Low risk
Ferrandina2008 [18]	Low risk	Low risk	High risk	Low risk	Low risk	Low risk	Low risk
Vergote2009 [19]	Low risk	Low risk	High risk	Low risk	Low risk	Low risk	Low risk
Colombo2012 [20]	Low risk	Low risk	High risk	Low risk	Low risk	Low risk	Low risk
Banerjee2018 [21]	Low risk	Low risk	High risk	Low risk	Low risk	Low risk	Low risk
Kaye2012 [22]	Low risk	Low risk	High risk	Low risk	Low risk	Low risk	Low risk

## Extraction of data

### Overall analysis of doublet regimens: PLD plus carbo vs. PAC plus carbo

**Fig. 2:**
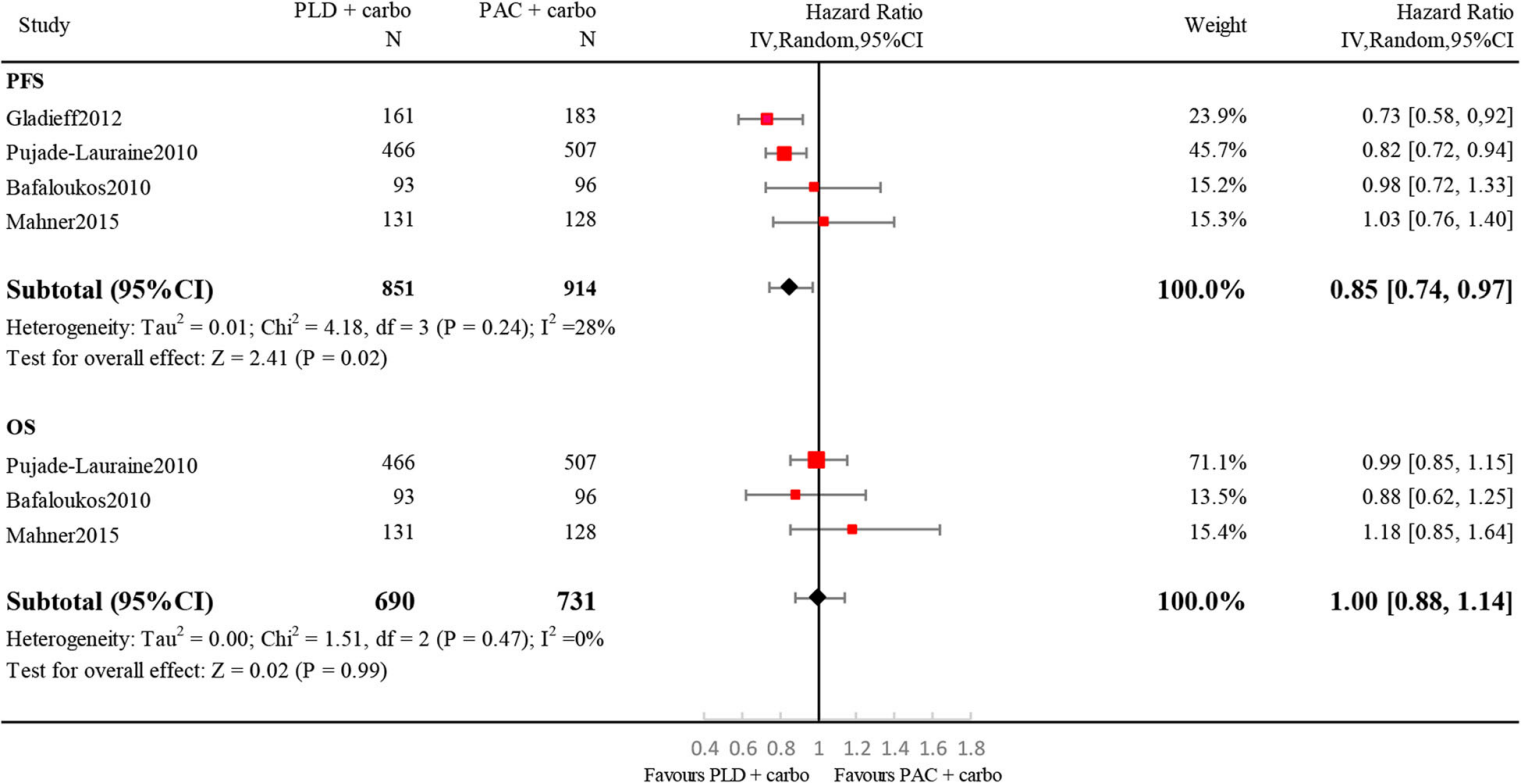
Forest plots of efficacy endpoints. Doublet regimens

PLD plus carbo was associated with a significant improvement in PFS (HR, 0.85; 95% CI, 0.74–0.97; I^2^ = 28%; *p* = 0.02), while OS was similar to the standard chemotherapy regimen PAC plus carbo (HR, 1.00; 95% CI, 0.88–1.14; I^2^ = 0%; *p* = 0.99) (Fig. 2).

**Fig. 3:**
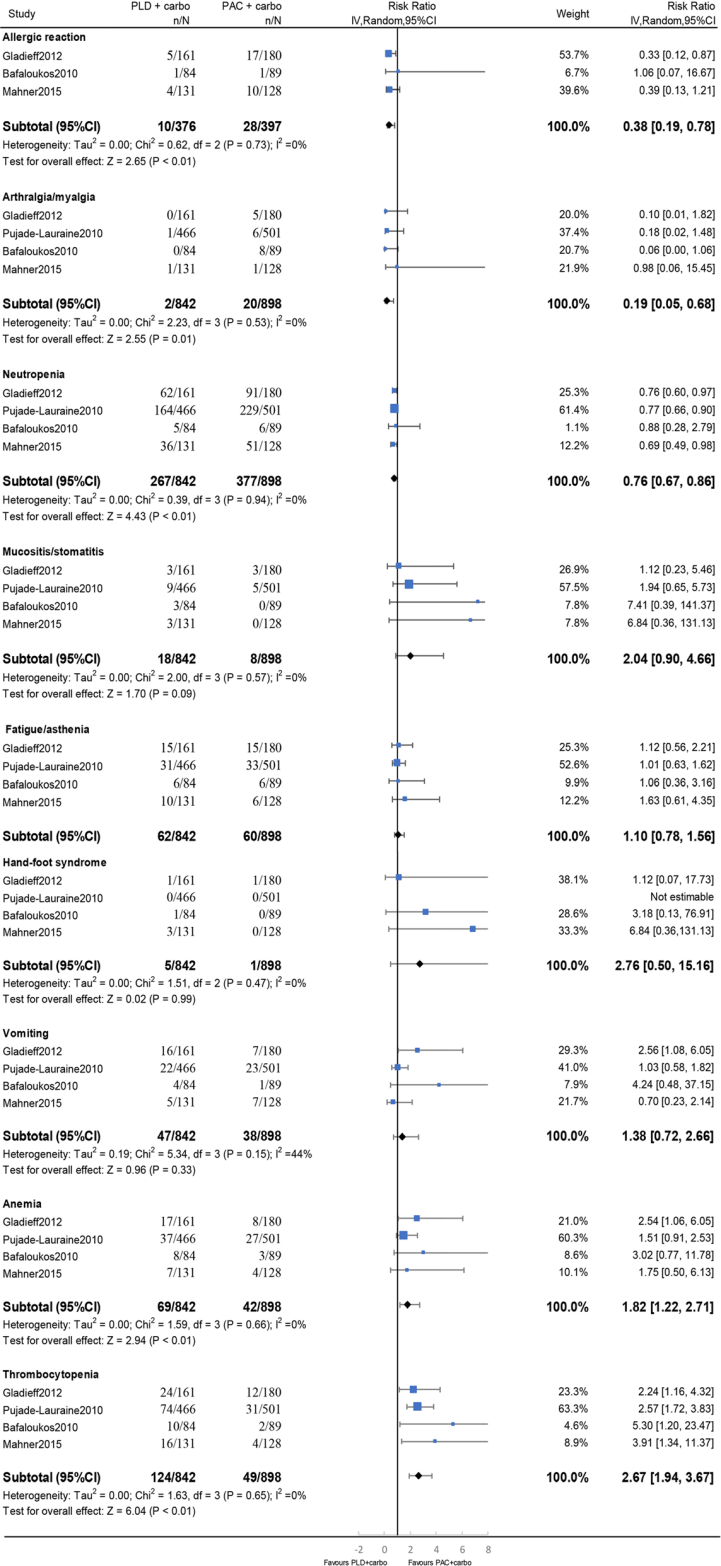
Forest plots of toxicity endpoints for the doublet regimens

With respect to grade 3–4 toxicities, PLD plus carbo was associated with a decreased risk of an allergic reaction (RR, 0.38; 95% CI, 0.19–0.78; I^2^ = 0%; *p* < 0.01), arthralgia/myalgia (RR, 0.19; 95% CI, 0.05–0.68; I^2^ = 0%; *p* = 0.01), and neutropenia (RR, 0.76; 95% CI, 0.67–0.86; I^2^ = 0%; *p* < 0.01). PLD plus carbo was also associated with an increased risk of anemia (RR, 1.82; 95% CI, 1.22–2.71; I^2^ = 0%; *p* < 0.01) and thrombocytopenia (RR,2.67; 95% CI,1.94–3.67; I^2^ = 0%; *p* < 0.01). There was no difference in the risk of fatigue/asthenia (RR, 1.10; 95% CI, 0.78–1.56; I^2^ = 0%; *p* = 0.57), mucositis/stomatitis (RR, 2.04; 95% CI, 0.90–4.66; I^2^ = 0%; *p* = 0.09), hand–foot syndrome (RR, 2.76; 95% CI, 0.50–15.16; I^2^ = 0%; *p* = 0.24), or vomiting (RR, 1.38; 95% CI, 0.72–2.66; I^2^ = 44%; *p* = 0.33) (Fig. 3).

### Overall analysis of monotherapy regimens: PLD vs. single agent

**Fig. 4:**
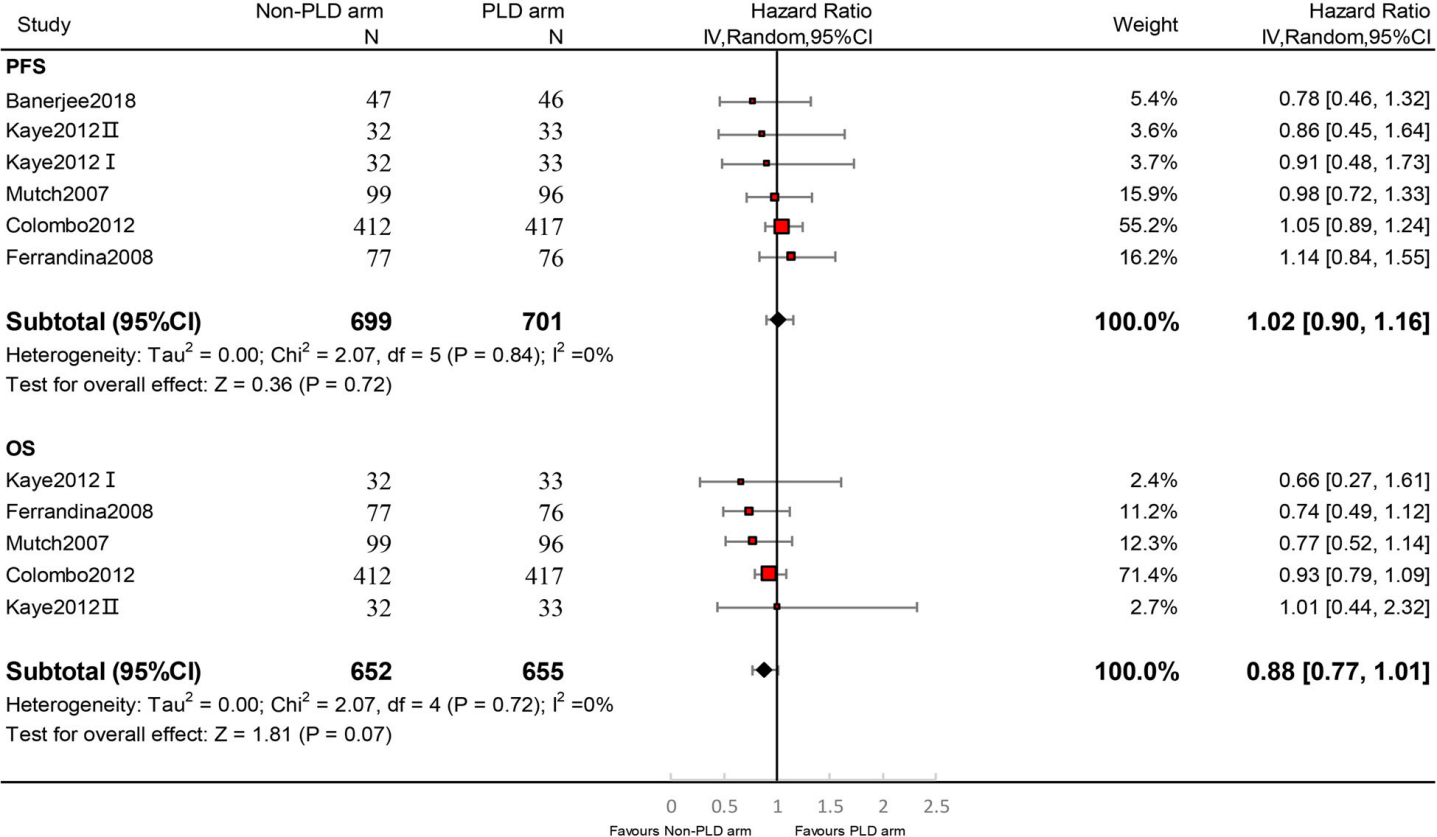
Forest plots of efficacy endpoints. Monotherapy regimens

PLD was similar in PFS (HR,1.02; 95% CI, 0.90–1.16; I^2^ = 0%; *p* = 0.72) and OS (HR, 0.88; 95% CI, 0.77–1.01; I^2^ = 0%; *p* = 0.07) to other single agents (Fig. 4).

**Fig. 5:**
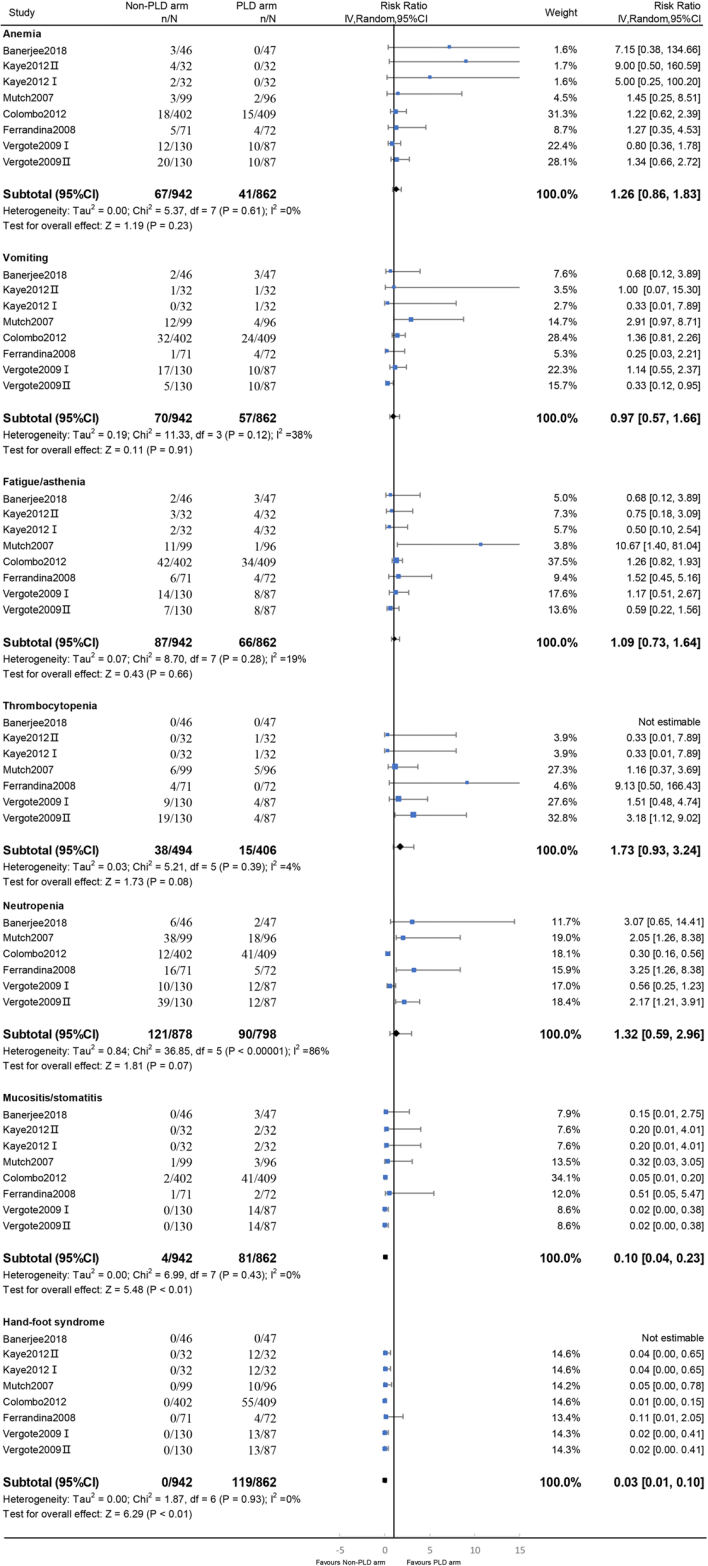
Forest plots of toxicity endpoints for the monotherapy

With respect to grade 3–4 toxicities, PLD was associated with a significantly increased risk of mucositis/stomatitis (RR, 0.10; 95% CI, 0.04–0.23; I^2^ = 0%; *p* < 0.01) and hand–foot syndrome (RR, 0.03; 95% CI, 0.01–0.09; I^2^ = 0%; *p* < 0.01) compared with the other monotherapies. There were no differences in the risks of anemia (RR, 1.26; 95% CI, 0.86–1.83; I^2^ = 0%; *p* = 0.23), vomiting (RR, 0.97; 95% CI, 0.57–1.66; I^2^ = 38%; *p* = 0.91), fatigue/asthenia (RR, 1.09; 95% CI, 0.73–1.64; I^2^ = 19%; *p* = 0.66), thrombocytopenia (RR, 1.73; 95% CI, 0.93–3.24; I^2^ = 4%; *p* = 0.08), or neutropenia (RR, 1.32; 95% CI, 0.59–2.96; I^2^ = 86%; *p* = 0.50) (Fig. 5).

### Subgroup analysis

We performed side-effect subgroup analysis with respect to neutropenia based upon the different drugs in the monotherapy regimens (I^2^ = 86%): one subgroup [18, 19] showed that canfosfamide and patupilone correlated with lower risk than PLD (RR, 0.39; 95% CI, 0.21–0.72; I^2^ = 33%; *p* < 0.01), while the other subgroup [16–18, 20] showed that gemcitabine, topotecan, Lifastuzumab vedotin (LIFA), and olaparib reflected higher risk than PLD (RR, 2.26; 95% CI, 1.61–3.17; I^2^ = 0%; *p* < 0.01). We then performed subgroup analysis for the differences in toxicity and side effects based on the different doses of PLD. In doublet regimens, we observed anemia at 30 mg/m^2^ vs. 45 mg/m^2^ PLD (I^2^ = 0%), and thrombocytopenia at 30 mg/m^2^ vs. 45 mg/m^2^ PLD (I^2^ = 0%). There was, however, no difference in the incidence of adverse reactions at the different doses of PLD. For monotherapy regimens, the incidence of mucositis/stomatitis was similar between 40 mg/m^2^ and 50 mg/m^2^ PLD (I^2^ = 60.5%), and hand–foot syndrome was similar between 40 mg/m^2^ and 50 mg/m^2^ PLD (I^2^ = 30.2%).

### Publication bias

To assess all studies with regard to PFS in potential publication bias, we used Egger’s linear regression test (*p* = 0.635), as well as Begg’s funnel plot (*p* = 0.592). The test results showed that this updated meta-analysis showed no significant publication bias (Supplementary Material 2).

## Discussion

To the best of our knowledge, the present study is the most recently updated meta-analysis with respect to the curative effects and side effects of PLD in recurrent ovarian cancer chemotherapy. Our results suggest that PLD is as effective or better in the treatment of recurrent ovarian cancer compared to other therapies. The secondary indicators showed that most patients tolerated the therapy well and manifested no serious adverse reactions.

### Doublet regimens

Our study results illustrated the superiority of platinum doublets of carbo plus PAC, carbo plus gemcitabine, and carbo plus PLD to single-agent platinum, and that carbo plus PLD was as effective as carbo plus PAC in women with highly sensitive and relapsed ovarian cancer [4, 22, 24, 25]. We therefore only selected and compared doublet regimens based on platinum in platinum-sensitive recurrent ovarian cancer. PLD plus carbo was superior in PFS without a change in OS. We found that of four doublet regimen trials, only the studies by Pujade-Lauraine2010 and Gladieff (2012) showed that PFS was prolonged in the PLD-plus-carbo group. In the Pujade-Lauraine study, 90% of the women received post-progression treatment, and the proportion of women in the PAC-plus-carbo arm who received PLD as post-study therapy (68%) was significantly higher than the proportion of women in the PLD-plus-carbo arm who received PAC (43%, *P* < 0.01), and this may have influenced the OS HR in the direction of the PAC-plus-carbo arm [11]. However, in the Gladieff study, OS was not assessed due to the fact that overall survival data were immature, such that there was no exact comparison between PFS and OS. Another perspective suggests the possibility that tumor cells that survive treatment with PLD plus carbo may be more aggressive or may be resistant to subsequent therapies. When the disease then recurs, it may progress more quickly or may be resistant to other therapies, thus negating any benefits on OS [10]. We also speculate that the study by Bafaloukos in 2010 (a phase-II study) did not have sufficient statistical power to assess OS, which may have affected the final results. The specific reasons for these disparate results remain unclear, and further research is therefore needed.

We compared PFS and OS based on different PLD doses, and did not observe any statistical significance between PLD at 30 mg/m^2^ every 4 weeks compared with PLD at 45 mg/m^2^ every 4 weeks. Therefore, we recommend that PLD at 30 mg/m^2^ every 4 weeks be used as the initial dosage in PLD-plus-carbo doublet regimens.

When we evaluated grade 2 or higher toxicities, we noted that PLD plus carbo was associated with a decreased risk of alopecia (RR, 0.09; 95% CI, 0.07–0.12; I^2^ = 0%; *p* < 0.01) and neuropathy (RR, 0.19; 95% CI, 0.14–0.27; I^2^ = 19%; *p* < 0.01) compared with PAC plus carbo. PLD plus carbo, however, was associated with an increased risk of mucositis/stomatitis (RR, 2.12; 95% CI,1.54–2.93; I^2^ = 0%; *p* < 0.01) and hand–foot syndrome (RR, 6.12; 95% CI, 3.84–9.76; I^2^ = 0%; *p* < 0.01).

Compared with grade 3–4 severe toxicities, both hand–foot syndrome and mucositis/stomatitis primarily arose with low-grade toxicities, and the patients’ adverse symptoms were mild. Both anemia and thrombocytopenia were principally associated with severe toxicities. Fortunately, the adverse incidence was not high (8.2 and 14.7%, respectively). We then laterally compared the incidence of side effects at two different PLD doses (grade 3–4 toxicity): for anemia, 30 mg/m^2^ vs. 45 mg/m^2^ PLD (8.0% vs. 9.5%, respectively), and for thrombocytopenia, 30 mg/m^2^ vs. 45 mg/m^2^ PLD (15.0% vs. 12.0%, respectively). These two adverse reactions did not show a significant dose-dependency for PLD, which may be because the combination with carbo reduced the toxic side effects of PLD.

Our updated meta-analysis results showed that PLD plus carbo provided a non-inferior survival rate and was well tolerated. Hence, PLD plus carbo emerged as a favorable option for platinum-sensitive patients in the recurrent setting.

### Single regimens

In platinum-resistant or -refractory recurrent ovarian cancer, PLD shows survival results similar to those of other single agents, and, thus, platinum-resistant women have been challenged with non-platinum drugs. One study showed that gemcitabine plus PLD was a very attractive combination given that it possessed different mechanisms of action and different toxicity profiles [26]; this combination did not reduce the individual effect of either agent, but rather increased the activity of the drugs in an additive fashion. This therapy was well tolerated by most platinum-resistant ovarian cancer patients, and patients with higher levels of baseline deoxycytidine kinase exhibited longer PFS. The usage recommended was 35 mg/m^2^ of PLD on day 1, and 1000 mg/m^2^ of gemcitabine on days 1 and 15 q4 weeks. However, as this was a phase-Ib study, it requires further exploration. Other investigators demonstrated that olaparib combined with PLD was well tolerated, but the combination did not result in a significant prolongation of PFS or OS in platinum-resistant or -refractory ovarian cancer [27]. The 2019 NCCN Guidelines showed that PLD plus bevacizumab constitutes a potential treatment option for patients with platinum-resistant recurrent ovarian cancer, and the 2020 NCCN Guidelines suggest that bevacizumab is effective in both platinum-sensitive and platinum-resistant recurrent ovarian cancer. Nevertheless, treatment of platinum-resistant or -refractory recurrent ovarian cancer as palliative care still necessitates more chemotherapy options.

The principal and common adverse effects of monotherapy PLD were mucositis/stomatitis and hand–foot syndrome. We laterally compared the incidence of side effects at two different PLD doses (grade 3–4 toxicity), and showed that mucositis/stomatitis (40 mg/m^2^ vs. 50 mg/m^2^) PLD (4.2% vs. 10.2%, respectively) was a dose-dependent side effect of PLD. At the same doses (3.4% vs 17.6%, respectively), the results showed that hand–foot syndrome was also a significant dose-dependent side effect of PLD.

Thus, our updated meta-analysis showed that PLD was well-tolerated at the 40 mg/m^2^ (lower-dose) regimen—which did not adversely affect survival compared with other single regimens—and confirmed PLD as a good choice for women in whom monotherapy was a treatment option.

### Strengths and limitations of PLD in the treatment of ovarian Cancer

The most concerning potential side effect of doxorubicin and PLD is often cited as congestive heart failure, and doxorubicin is in fact closely associated with congestive heart failure. PLD’s parent drug is doxorubicin, but PLD can effectively reduce cardiac toxicity. Studies show that PLD reduced the incidence of cardiotoxicity five-to-six fold even at doses ≥500 mg/m^2^. This is because of pegylation, which coats the liposome with a hydrophilic protective coating, and allows the drug to remain in circulation for a prolonged time due to its ability to evade immunologic elimination. Both lower plasma levels and improved ability to target tumor tissue allow for the sparing of cardiac toxicity with PLD [28]. One study depicted no significant incidence of cardiotoxicity (as defined by 2D strain on 3D left-ventricular ejection fraction), even with high cumulative doses of PLD up to 2500 mg, and therefore long-term use appears safe [29]. Thus PLD exerts a cardioprotective effect and is more beneficial for patients with poor heart function and for elderly patients.

Contemporary studies have shown that prolonged treatment with PLD is associated with the development of secondary squamous cell carcinoma of the oral mucosa in a number of case reports [9]. In another trial, we showed that the cumulative doses of PLD in our patients prior to the development of squamous cell carcinoma were 1350 mg/m ^2^ and 1142 mg/m ^2^, respectively, and that it was necessary to reduce the dose, prolong the administration, and provide regular oral-cavity examinations along with complete skin examinations [30]. We recommend using a PLD dose as low as possible, and to prolong the administration so as to reduce the incidence of hand–foot syndrome—thereby reducing the incidence of secondary squamous cell carcinoma of the oral mucosa.

## Conclusions

PLD plus carbo for platinum-sensitive disease produced a better PFS than standard-regimen PAC plus carbo and was well tolerated. However, there was no difference in overall survival. The findings of this meta-analysis support the continued use of PLD plus carbo as first-line chemotherapy for platinum-sensitive recurrent ovarian cancer, and PLD at 30 mg/m^2^ every 4 weeks can be used as the initial dose. As a single-agent therapy, PLD manifested survival similar to other agents and was well tolerated. The findings of this meta-analysis support the continued use of PLD monotherapy as first-line chemotherapy for platinum-resistant or -refractory recurrent ovarian cancer, and we recommend using PLD at a dose of 40 mg/m^2^ every 4 weeks as the initial dose.

## Supplementary Information

**Additional file 1.**

**Additional file 2.**

## Data Availability

The dataset used or analyzed in this study is available from the corresponding author upon reasonable request.

## Abbreviations

NCCN: National comprehensive cancer network
PLD: Pegylated liposomal doxorubicin
PAC: Paclitaxel
Carbo: Carboplatin
LIFA: Lifastuzumab vedotin
OS: Overall survival
PFS: Progression-free survival
HR: Hazard ratios
RR: Risk ratios
95% Cl: 95% Confidence interval.
