# Supplementary material for: The impact of Pegylated liposomal doxorubicin in recurrent ovarian cancer: an updated meta-analysis of randomized clinical trials
Source: J Ovarian Res. 2021 Mar 9;14:42. doi: 10.1186/s13048-021-00790-4 (PMC7945320; doi:10.1186/s13048-021-00790-4)
Supplement: Supplementary file 1 — Additional file 1. [file 13048_2021_790_MOESM1_ESM.docx]

S.1 Search Strategies

**Pubmed**

Search (((("liposomal doxorubicin" [Supplementary Concept]) OR (((((Pegylated Liposomal Doxorubicin[Title/Abstract]) OR Caelyx[Title/Abstract]) OR Lipodox[Title/Abstract]) OR DOX-SL[Title/Abstract]) OR Doxil[Title/Abstract]))) AND (("Ovarian Neoplasms"[Mesh]) OR (((((((((((Neoplasm, Ovarian[Title/Abstract]) OR Ovarian Neoplasm[Title/Abstract]) OR Neoplasm, Ovary[Title/Abstract]) OR Ovary Neoplasm[Title/Abstract]) OR Neoplasms, Ovarian[Title/Abstract]) OR Ovary Cancer[Title/Abstract]) OR Cancer, Ovary[Title/Abstract]) OR Ovarian Cancer[Title/Abstract]) OR Cancer, Ovarian[Title/Abstract]) OR Cancer of Ovary[Title/Abstract]) OR Cancer of the Ovary[Title/Abstract]))) AND ((("Randomized Controlled Trials as Topic"[Mesh]) OR (((Clinical Trials, Randomized[Title/Abstract]) OR Trials, Randomized Clinical[Title/Abstract]) OR Controlled Clinical Trials, Randomized[Title/Abstract])) OR "Randomized Controlled Trial" [Publication Type])

**Embase**

Search (('doxorubicin'/exp) OR ('liposomal doxorubicin':ti,ab,kw OR 'pegylated liposomal doxorubicin':ti,ab,kw OR caelyx:ti,ab,kw OR lipodox:ti,ab,kw OR 'dox sl':ti,ab,kw OR doxil:ti,ab,kw)) AND (('ovary tumor'/exp) OR ('ovarian neoplasms':ti,ab,kw OR 'neoplasm, ovarian':ti,ab,kw OR 'ovarian neoplasm':ti,ab,kw OR 'neoplasm,ovary':ti,ab,kw OR 'ovary neoplasm':ti,ab,kw OR 'neoplasms, ovarian':ti,ab,kw OR 'ovary cancer':ti,ab,kw OR 'cancer,ovary':ti,ab,kw OR 'ovarian cancer':ti,ab,kw OR 'cancer,ovarian':ti,ab,kw OR 'cancer of ovary':ti,ab,kw OR 'cancer of the ovary':ti,ab,kw)) AND (('randomized controlled trial(topic)'/exp) OR ('clinical trials,randomized':ti,ab,kw OR 'trials,randomized clinical':ti,ab,kw OR 'controlled clinical trials,randomized':ti,ab,kw))

**Web of science**

Search (TS=((liposomal doxorubicin) OR (Pegylated Liposomal Doxorubicin) OR (Caelyx) OR (Lipodox) OR (DOX-SL) OR (Doxil))) AND (TS=((Ovarian Neoplasm) OR (Neoplasm, Ovarian) OR (Ovarian Neoplasm) OR (Neoplasm,Ovary) OR (Ovary Neoplasm) OR (Neoplasms, Ovarian) OR (Ovary Cancer) OR (Cancer, Ovary) OR (Ovarian Cancer) OR (Cancer, Ovarian) OR (Cancer of Ovary) OR (Cancer of the Ovary)))AND(TS= ((Randomized Controlled Trials) OR (Clinical Trials, Randomized) OR (Trials, Randomized Clinical) OR (Controlled Clinical Trials,Randomized)))

**Cochrane Library**

Search (((MeSH descriptor:[Ovarian Neoplasms] explode all trees) OR ((Neoplasm,Ovarian):ti,ab,kw OR (Ovarian Neoplasm):ti,ab,kw OR (Neoplasm ,Ovary):ti,ab,kw OR (Ovary Neoplasm):ti,ab,kw OR (Neoplasm,Ovarian):ti,ab,kw) OR ((Ovary Cancer):ti,ab,kw OR (Cancer,Ovary):ti,ab,kw OR (Ovarian Cancer):ti,ab,kw OR (Cancer, Ovarian):ti,ab,kw OR (Cancer of Ovary):ti,ab,kw) OR((Cancer of the Ovary):ti,ab,kw))) AND (((liposomal doxorubicin):ti,ab,kw OR (Pegylated Liposomal Doxorubicin):ti,ab,kw OR (Caelyx):ti,ab,kw OR (lipodox):ti,ab,kw OR (DOX-SL):ti,ab,kw) OR ((Doxil):ti,ab,kw))
