# Supplementary figures and images for: The impact of Pegylated liposomal doxorubicin in recurrent ovarian cancer: an updated meta-analysis of randomized clinical trials
Source: J Ovarian Res. 2021 Mar 9;14:42. doi: 10.1186/s13048-021-00790-4 (PMC7945320; doi:10.1186/s13048-021-00790-4)

Egger's publication bias plot

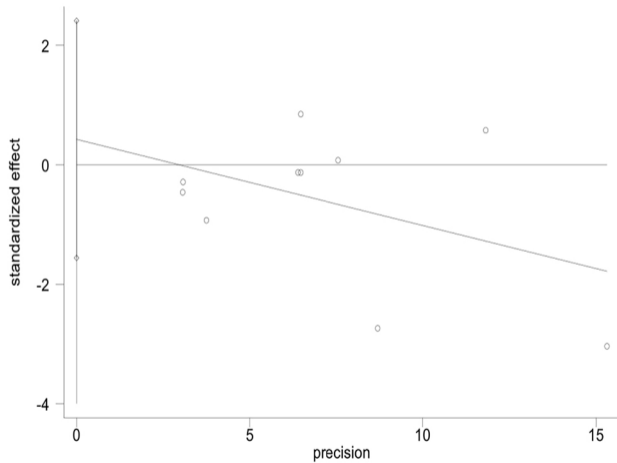

Begg's funnel plot with pseudo 95% confidence limits

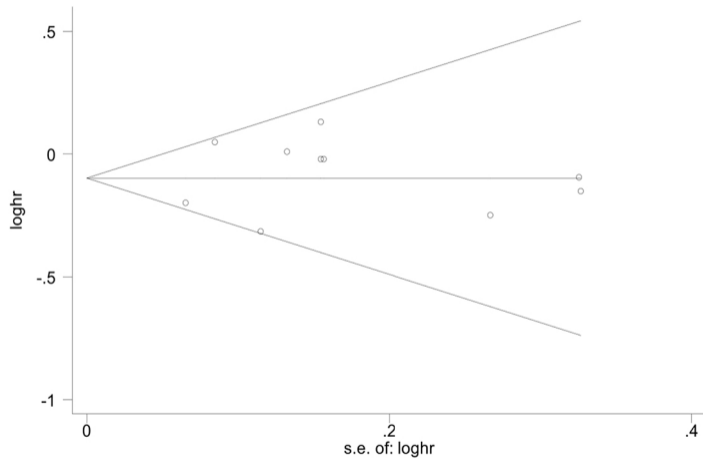

Supplement: Supplementary file 2 — Additional file 2. [file 13048_2021_790_MOESM2_ESM.pdf]
